# Circulating microbial RNA and health

**DOI:** 10.1038/srep16814

**Published:** 2015-11-18

**Authors:** Ross Ka-Kit Leung, Ying-Kit Wu

**Affiliations:** 1 Stanley Ho Centre for Emerging Infectious Diseases, The Chinese University of Hong Kong, Shatin, NT, Hong Kong, The People’s Republic of China; 2 Division of Genomics and Bioinformatics, CUHK-BGI Innovation Institute of Trans-omics, The Chinese University of Hong Kong Shatin, N.T., Hong Kong, The People’s Republic of China.

## Abstract

Measurement of health indicators in the blood is a commonly performed diagnostic procedure. Two blood studies one involving extended observations on the health of an individual by integrative Personal Omics Profiling (iPOP), and the other tracking the impact of Left Ventricular Assist Device (LVAD) placement on nine heart failure patients were examined for the association of change in health status with change in microbial RNA species. Decrease in RNA expression ratios of human to bacteria and viruses accompanying deteriorated conditions was evident in both studies. Despite large between-subject variations in bacterial composition before LVAD implantation among all the patients, on day 180 after the implantation they manifested apparent between-subject bacterial similarity. In the iPOP study three periods, namely, pre-respiratory syncytial virus (RSV) infection with normal blood glucose level, RSV infection with normal blood glucose level, and post-RSV infection with high blood glucose level could be defined. The upsurge of Enterobacteria phage PhiX 174 *sensu lato* and *Escherichia coli* gene expression, in which membrane transporters, membrane receptors for environment signalling, carbohydrate catabolic genes and carbohydrate-active enzymes were enriched only throughout the second period, which suggests a potentially overlooked microbial response to or modulation of the host blood glucose level.

There is a long history of detection of previously known microbial RNA in blood and blood products in clinics using RT-PCR, pan-viral and pan-microbial microarrays. To understand the systematic picture of microbial composition and to probe potential interactions, unbiased deep sequencing RNA-seq is a possible solution. DNA sequencing technology improvements and an arsenal of massively parallel sequencing (MPS) data processing methods and tools^1^,^2^,^3^,^4^,^5^,^6^,^7^,^8^ have opened up opportunities for “omics” research and services. Integrating expertise in pharmacogenomics, pathology, medical genetics, statistics and bioinformatics, clinically relevant interpretations for a patient’s genome have also been made^9^. In an omics profiling for an individual over a 14-month period (denoted as iPOP hereafter), Chen *et al.* observed that after RSV infection, 1) glucose levels elevated and extended for at least 90 days, 2) some genes involved in insulin signaling response and immune function exhibited downward trend of expression, and 3) cytokine levels elevated at day 12 after the infection without any apparent explanation^10^. Another survey that tracked nine heart failure patients before and after implanting continuous-flow left ventricular assist devices (LVADs) only focused on human gene expressions that promote a healthy immune response^11^, without exploring the dynamics of microbiome that might be associated with the change in post-operative health.

Scientific evidence is beginning to suggest that microbial footprints are more pervasive than imagined and microbiome were identified in body fluids^12^,^13^. Blood microbiome has been associated with infectious and even chronic diseases, including rheumatoid arthritis, anemia, type 2 diabetes, neurological and cardiovascular diseases (reviewed by Potgieter *et al.*^14^). Potgieter *et al.* also suggested that the origin of the blood microbiome is a dormant form from the gut and argued that contamination alone cannot explain considerable observations made in the literature^14^. Blood microbiome was not studied in the two large-scale microbiome initiatives^15^,^16^. Even in the second phase of Human Microbiome Project, the Integrative Human Microbiome Project^17^, blood microbiome is still missing on the agenda.

The scope of human health has widened after numerous reports on the role of microbiota in human health and diseases. From the two initiatives it is now evident that both the cells and genes of microbiota outnumber their human host’s and some of which even contribute to human survival. Although microbiota can be dynamically changing and vary significantly between than within individuals, the presence of “essential” microbial metabolic activities matter more^18^,^19^. Endogenous microbiota also play an important role in protecting their host from infection, even in the eyes by driving the maturation of CD4^+^T cells^20^ or development of diabetes^21^. At the other extreme, when immune selection is weak or absent, changes in microbiome in throat and possibly originated from gut were associated with change in health status^22^,^23^. Microbiota may also exert long-distance effects by the presence of their components, for examples, encapsulated RNA^24^ and other small molecules (reviewed by Potgieter *et al.*^14^) in the blood. Wang *et al.* conducted an extensive study of evaluating the content of non-human RNA in blood components by computer simulation, biochemical, transfection, immunoprecipitation and expression experiments and concluded the existence of a significant fraction of circulating exogenous RNA, some of which may mediate cellular gene expression^24^. The concepts underlying the human microbiome and its relation to human health, disease and potential microbiome restorative therapies were reviewed by Khanna and Tosh^25^ and Round and Mazmanian^26^.

The two aforementioned multiple-time-point studies employed MPS for RNA analysis, also known as RNA sequencing (RNA-seq). The first study collected peripheral blood mononuclear cells (PBMCs) at 20 time points: roughly partitioned into periods of after human rhinovirus (HRV) infection and after respiratory syncytial virus (RSV) infection^10^. Although most PBMCs are lymphocytes, in which relatively few (if any) microbial nucleic acids can be identified, there are monocytes which constitute about 10% of the PBMC population in a healthy individual^27^. These monocytes, alongside differentiated macrophages and dendritic cells may contain microbial nucleic acids indicative of health status. The second study (denoted as LVAD hereafter) analyzed blood RNA samples drawn from the heart failure patients at three time points: pre-LVAD implantation, one-week and 180-day post-implantation^11^. These two studies together provide valuable resources for evaluating within- and between-subject metatranscriptomic variations for health monitoring and etiology hypotheses.

## Results

Our analytical procedures were able to reveal the expression of all the 11 genes of PhiX 174 *sensu lato* with even and deep coverage for Lappalainen, T. *et al.*’s sequencing data^28^ (Supplemental Table 1). Less than 0.01% of the total simulated sequencing reads with 0 or 2 mismatch incorporation could not be mapped onto human, bovine, mouse and yeast reference RNA and these unmapped reads were mostly poly T or poly A sequences. For those that could be mapped, they were all mapped to the correct domain reference (Supplemental Table 1). For the iPOP and LVAD datasets used in this study, the percentage of uniqueness for the sequences assigned to non-human species was 83.92 ± 12.81% (Supplemental Table 1).

**Figure 1:**
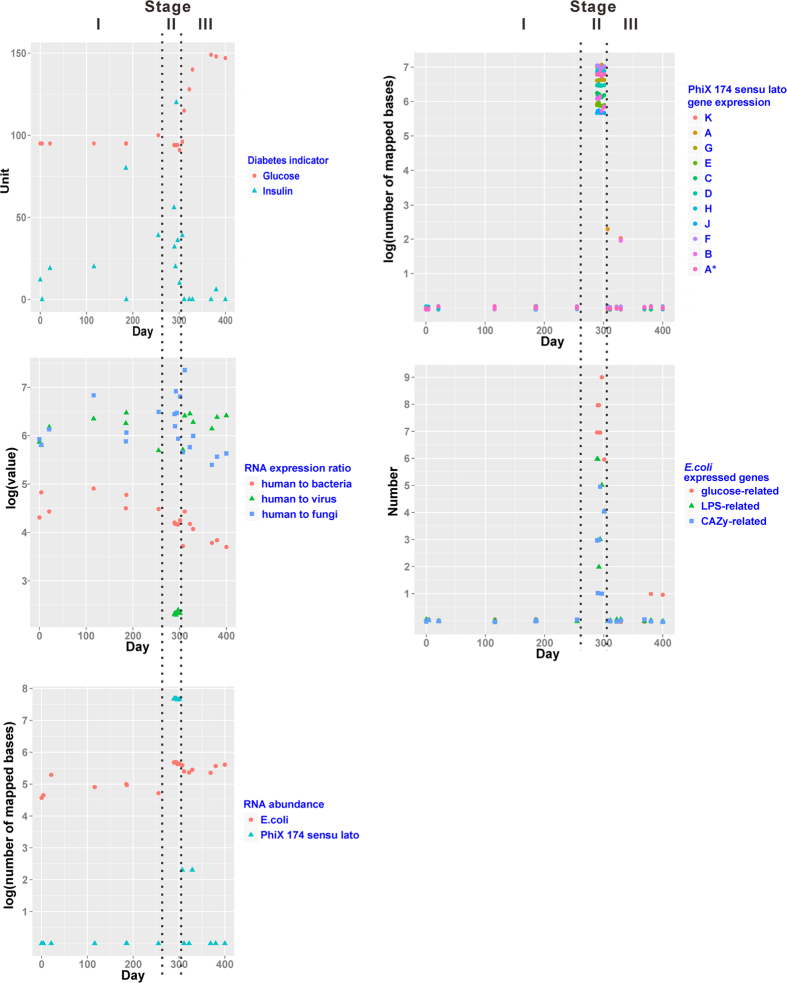
Plots of hyperglycemia indicators, RNA expression ratios of human to microbiome, relative RNA abundance and gene expression level over the 14-month iPOP study. LPS: lipopolysaccharide, CAZy: Carbohydrate-Active Enzymes. Stage I: after HRV but before RSV infection, Stage II: after RSV infection until the last time point before the onset of high blood glucose. Stage III: after the onset of hyperglycemia.

An overview about the basic information, mapping statistics, and RNA expression ratios of human to bacteria (RNA human/bact), human to viruses (RNA human/virus) and human to fungi (RNA human/fungi) at different time points of the iPOP and LVAD studies is summarized in Supplemental Table 2. The RNA human/fungi was constantly high (>105) under different settings and therefore it was not further investigated (Fig. 1). The association of microbiome with health status is presented below at ascending level of details: 1) RNA expression ratios of human to bacteria and viruses; 2) community composition; and 3) gene catalogue.

### RNA expression ratios of human to bacteria and viruses

The decrease in RNA human/bact over the whole iPOP study period was associated with two events: RSV infection and onset of increase in blood glucose level. Prior to RSV infection, RNA human/bact fluctuated at a relatively high level (median = 31,514, Interquartile range = 40,277) (Supplemental Table 2 and Fig. 1). After RSV infection, the RNA human/bact steadily maintained at about 15,000 (Interquartile range = 1,817). RNA human/virus reached its lowest during this period (median = 214, Interquartile range = 24). When the blood glucose level started to rise on day 307, RNA human/virus returned to the previous level but RNA human/bact appeared to further drop, though statistically insignificantly. The comparison of RNA human/bact and RNA human/virus between the periods of day 0 until the last time point before RSV infection (day 0 to day 255), day RSV infection detected until the last time point before the onset of high blood glucose (day 289 to day 301) and after the onset of high blood glucose (day 307 to day 400) by post-hoc test using Mann-Whitney tests with Bonferroni correction is summarized in Supplemental Table 3. To evaluate whether the high RNA human/bact ratios observed for five consecutive time points after RSV infection was due to artifacts or contamination, we conducted a permutation test of 20 time points to count the occurrence of consecutive time points after random shuffling. Permutation results suggested that five consecutive time points displaying high RNA human/bact ratios just after RSV infection is not common (Supplemental Table 4). In the LVAD study, the changes of RNA human/bact and RNA human/virus (Supplemental Fig. 1) agreed with the prior observations that a healthy immune response in patients with heart failure had only been partially restored six months following LVAD implantation^11^.

### Community composition

**Figure 2:**
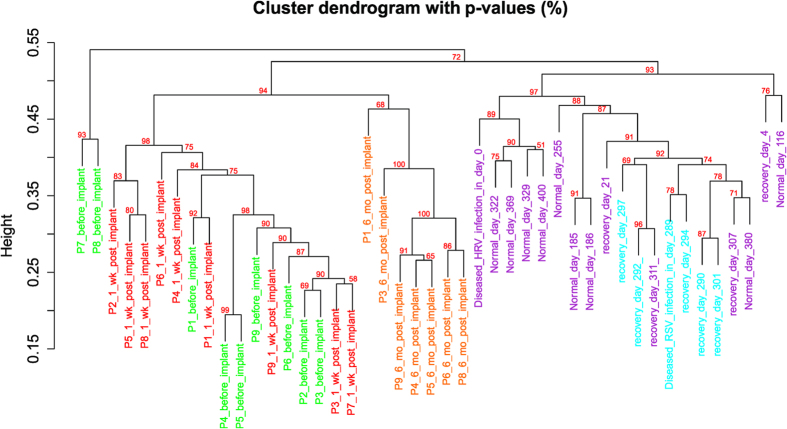
Hierarchical clustering of bacterial RNA at genus level from the 9 + 1 individuals at different time points respectively from the LVAD and iPOP studies. Green: pre-LVAD implantation, Red: 1 week post implantation, Orange: 180 days post implantation, Cyan: RSV infection period, Purple: non-RSV infection period. P1-P9: patients 1–9, wk: week, mo: month.

**Figure 3:**
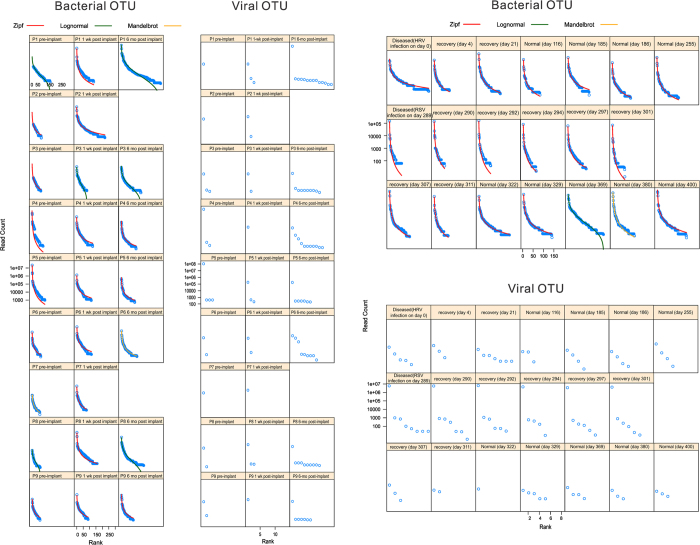
Rank abundance curves based on bacterial and viral OTUs. The viral communities in iPOP and LVAD study was not diverse enough for niche model fitting. Since no pre-emption model was fitted and Zipf-Mandelbrot model was of minority in the bacterial communities, there were few non-linear components and one or two genera usually dominated. P1-P9: patients 1–9, wk: week, mo: month.

Although within-subject similarity was obvious in the individual recruited in the iPOP study, within-subject similarity was violated for the patients in the LVAD study. The bacterial gene expression profiles among individuals were synchronized at 180 days post implantation (Fig. 2). The cluster analysis suggests that batch effects were insignificant, or otherwise distinct green, red and orange clusters or branches would have been observed. The “synchronization” was due to introduction of new bacterial RNA species and independent of total number of bacterial RNA. For patients 4 and 5, the total number actually dropped but the bacterial diversity increased (Supplemental Table 5). All the nine patients manifested shallower rank abundance curves after operation, the longer the interval the more obvious (Fig. 3). Of universal presence in all the nine patients at all the three time points, only the RNA species from *Ralstonia*, but not other potential nosocomial infectious agents from the genera of *Micrococcus, Burkholderia, Sphingobium, Cupriavidus, Streptococcus, Pseudomonas, Staphylococcus* and *Escherichia* increased significantly one-week post-operation in patients 2, 4, 6, 7 and 8 (but it also dropped significantly in patient 3). Mapping results validated by BLAST search against the nucleotide sequence database nt showed that the *Ralstonia* RNA species were most likely from *Ralstonia (R.) pickettii* (Supplemental Table 6). The *Ralstonia* RNA level returned to previous values on day 180 post-implantation.

Steep gradients of rank abundance curves were observed for the individual once infected by RSV (days 289–301) (Fig. 3), due to the explosive expansion of RNA species from potential gut flora, including those under the genera *Shigella*, *Escherichia*, *Klebsiella, Enterobacter* and *Salmonella* (approximately 12, 7, 4, 3 and 2 folds respectively) compared to the period prior to the infection (days 0–255) (Supplemental Table 6). The same five bacterial genera RNA species were also significantly enriched contemporarily in patient 1 on 180 days post-implantation and in patients 4 and 5 before implantation. We examined the mapped *Escherichia* RNA at species level in the iPOP and LVAD studies and found that at least 96% of them were likely from *Escherichia (E.) coli*. The *E. coli* mapping result was also supported by best BLAST matches of *E. coli* against nt to the mapped contigs, minimizing the chance of random mapping by BWA (Supplemental Table 6). The number of viral RNA species followed the same trend as that observed in the bacterial RNA dynamics in both studies (Fig. 3).

We noticed that Enterobacteria phage PhiX 174 *sensu lato* comprised more than 99% of the entire virus identified from days 289 to 301. In contrast, no RSV was identified, as expected. Trace amount of RNA from the phage was first identified on day 255, followed by an upsurge until the onset of high blood glucose level (Fig. 1). In fact, RNA from Enterobacteria phage PRD1, Enterobacteria phage HK97, Enterobacteria phage GA, human adenovirus C and human herpesvirus 4 were also only detected during this period, though their abundance compared to that of PhiX 174 *sensu lato* was negligible. Steady and similar expression from all the eleven PhiX 174 *sensu lato* genes was identified (Supplemental Table 6); comparable to those from three independent PhiX RNA-seq datasets (see Methods and Supplemental Table 1). PhiX 174 *sensu lato* also comprised more than 99% of the entire virus identified in patient 1 180 days post-implant and in patients 4 and 5 before implantation, and more than 89% in the other patients excepting patient 6 at 180 days after LVAD implantation (63.9%).

### Gene catalogue

To explore possible mechanisms driving subsequent rising blood glucose after the over-dominance of PhiX 174 *sensu lato* and *E. coli* gene expression after RSV infection, we determined gene functional category enrichment by the DAVID Gene Functional Classification Tool^8^. During the periods of pre-RSV infection and high blood glucose level, only ribosomal protein transcripts were identified to be statistically significant (False Discovery Rate (FDR) < 0.05, Supplemental Table 7). Strikingly a large number of gene functional categories enriched during RSV infection was identified, including membrane transporters; membrane receptors for environment signaling; and carbohydrate catabolic process. We were particularly interested in the genes that might have also accounted for the onset of high blood glucose level. The numbers of CAZyme (Supplemental Table 7), glucose- and lipopolysaccharide (LPS)-related gene expression (Supplemental Table 8) were the highest during the RSV infection period. Although RNA human/bact were amongst the lowest on days 400, 307, 369 and 380, the three aforementioned gene categories were not enriched (Fig. 1).

The simultaneous enrichment in Enterobacteria phage PhiX 174 *sensu lato* and *E. coli* glucose-related gene expression was also identified in the LVAD study (patient 1 180 days after LVAD treatment and in patients 4 and 5 pre-implantation). The numbers of corresponding bacterial glucose-related gene were 51, 45 and 34; where in other cases were almost zero (Supplemental Table 6). Similar results of LPS and CAZy matches and gene functional category enrichment were also obtained (Supplemental Tables 7–8).

No particular gene functional category except ribosomal protein was enhanced in *R. pickettii* expressed genes identified in the LVAD patients at one-week post-implantation (Supplemental Table 9). The ribosomal protein enrichment pattern tightly followed the change of total *R. pickettii* RNA species (Supplemental Table 6).

## Discussion

Our findings facilitate the exploration of best practice guidelines in prophylaxis and health management by examining microbiome RNA expression at different levels of detail in blood/PBMC samples. The first level, RNA expression ratio of human to microbiome, requires the minimal specialized skills to derive and is the most intuitive for interpretation. The change in the ratio was associated with deteriorating health status in the iPOP and LVAD studies. The second and third levels, community composition and gene catalogue can serve useful clues to guide investigation of the composition of potential health modulators and effecting mechanisms.

Although human gene expression signatures of heart failure were characterized^11^,^29^,^30^, surprisingly there are few, if any, studies concurrently characterizing any microbiome altered after LVAD implantation, despite that LVAD support is associated with infections^31^. Reported median and maximum hospital length of stay of LVAD implant patients were 20 and 90 days^32^, therefore the significantly increased diversity observed at 180 days post-implantation unlikely represented newly acquired nosocomial infections. A plausible explanation is nutrient availability^33^, which facilitates the colonization of more diverse types of bacteria, instead of only enabling the survival of oligotrophs, for example, the nosocomial infectious agent *R. pickettii*. Our findings indeed showed that RNA from the aerobic, motile *R. pickettii* increased significantly in five patients one week after the operation but returned to a previous level on day 180 after implantation. The decrease in the RNA proportion of motile bacteria might also imply loss of niche when nutrients become more available. In addition to Ralstonia, RNA species from common nosocomial infectious agents Pseudomonas and Staphylococcus were also universally present in all the patients over the three time points. The species from these two genera are also the most common causative pathogens to LVAD patients^31^. Nevertheless, their RNA species were not significantly enriched in any single patient. In summary, taking the microbial components into consideration of the specific differential gene expressions involved in healthy immune response after LVAD placement identified by Mitchell *et al.*^11^ is important because the blood transcriptome dynamics might actually be the outcome of complex interplay between host response to subclinical infections and micro-organisms adapting varying environmental conditions. To optimize the response to LVAD^34^, management of (subclinical) infections^35^ may play a significant role in future care of patients with end stage heart failure.

Previously unexplored connections between the onset of elevated glucose levels and microbiome were identified. The low RNA human/bact and RNA human/virus alone sufficed to reveal the RSV infection status. When bacterial community was examined, the relative abundance of *E. coli* RNA species was found to increase up to almost 80% after RSV infection (Supplemental Table 6) but *E. coli* is present at no more than one percent abundance of gut flora in healthy human stool microbiomes^18^. High blood glucose level was observed only beginning from day 307 but the gene enrichment in carbohydrate metabolism only lasted until day 301, exactly the day when an unexplained cytokine spike was identified^10^. Interestingly, the disappearance in gene enrichment was accompanied by significant decrease of PhiX 174 *sensu lato* RNA. The same co-existence of the phage and carbohydrate metabolism was also observed in the LVAD study, suggesting the functional implications of the phage in the *E. coli* gene expression. In fact, PhiX 174 *sensu lato* A* gene can inhibit DNA replication and cell division but not beta-galactosidase^36^ and possibly other genes in *E. coli*. The phage was likely to be active during the RSV infection period because steady and comparable expression of all the eleven PhiX 174 *sensu lato* genes was identified (Supplemental Table 6). Lipopolysaccharide-related gene expression of *E. coli* (Supplemental Table 6, Fig. 1) might also facilitate PhiX 174 *sensu lato* infection^37^. Moreover, naked RNA is prone to degradation^38^, probably due to the ubiquitous RNAse in the blood^39^. Chen *et al.* identified that immune cell and insulin signaling had been perturbed to some extent during the RSV infection and high glucose states of their study subject^10^. Whether the PhiX 174 *sensu lato-Escherichia coli*-blood glucose axis was a cause, a consequence or an independent event of the perturbation warrants further investigations.

The setting of this study carried several limitations. Firstly, the metatranscriptome and metagenome may not be related directly^40^. Nevertheless, once quantitative relationship between metagenomic composition and human health is established, cheaper, faster and more convenient detection method such as gel electrophoresis of 16S rRNA amplicons can then be performed, an approach adopted to investigate the association of the gut microbiota profile with insulin action in humans^41^. Secondly, this study does not allow the inference of causality. While time-series data would be ideal to reveal this, a full-fledged analysis was limited by data availability. We attempted to collect healthy controls from a public data repository. For those datasets with accompanying publications in the Sequence Read Archive, different sample preparation methods have created irreversible data loss. These include filtering^42^, isolation and purification of intracellular RNA only^43^, gene-specific amplification^44^ or exome capture^45^. Although the RNA expression ratios of human to bacteria among different studies were still larger than one, the variations were very large (Supplemental Table 10). Indeed, between-subject variations were also reported to be large in Human Microbiome Project^18^. This was also the reason why the two studies with within-subject blood microbiome at different time points were chosen. Thirdly, the main challenge to identify a good normalization method for meta-transcriptomics is complexity. A typical transcriptome study deals with the total mRNA of a single organism under two or more conditions. In a meta-transcriptomics study, there are multiple organisms which can have very different genes in character and quantity to be measured of their expression. Recently, there has been some development in normalizing RNA-sequencing data from samples with varying mRNA levels^46^ and using mixtures of biological samples as process controls^47^. However, the former still concerns only a single organism and the later assumes well defined mixture, which is unavailable in this study. Therefore the methods in the two aforementioned studies cannot be directly applied to this study. Removing unwanted variation by performing factor analysis on suitable sets of control genes^48^ is also difficult, because how to identify the set of control genes representing multiple organisms warrants rigorous research efforts. Finally, false discovery rates for enrichment across species were not carried out. For meta-transcriptomics the calculation again demands special handling like that of normalization. In this study, there is no well-defined boundary or test-control comparison. When the number of combinations increases, the total number of hypothesis tests that are to be performed also increases accordingly. Nevertheless, there is a need to develop a rigorous assessment method to control false discoveries in future.

In this work, we put forward the concept of comparing circulating microbial RNA can provide insights into the possible mechanisms/manifestations of pathological conditions and therefore we only reported the most apparent differences (for example, see Fig. 1), in which the differential gene expression was up to 10 to 107 fold. We wish our findings would on one hand inspire experts from mathematics, statistics, computer science or other physical sciences to develop rigorous assessment methods and on the other hand generate attention in the scientific community so that more longitudinal studies (like iHMP) would be carried out to track the dynamics of blood microbiome and health. Plummeting sequencing cost and accumulated knowledge of microbiome can be exploited to add value to the relatively readily available blood sampling for acquiring rich and often interwoven molecular and physiological information, which can be analyzed for health status and health dynamics monitoring.

## Methods

The methodological details of sample preparation can be found in the original published papers. Briefly, in the integrative personal omics profile (iPOP) study^10^, the molecular and physiological information derived from harvested peripheral blood mononuclear cells (PBMC) of a generally healthy individual was monitored at 20 time points over a span of 14 months. In the second study^11^, each of nine end stage heart failure patients was implanted with a left ventricular assist device (LVAD) and subjected to whole blood collection before and after implantation. Sequencing control lane data of PhiX 174 RNA^28^ were used as data quality control in this study. RNA-seq datasets of these three studies were retrieved from NCBI Sequence Read Archive (SRA). The SRA accession numbers for the iPOP, LVAD and PhiX datasets are from SRR353635 to SRR353654; SRR846935 to SRR846959 and ERR011381, ERR011423 and ERR011436 respectively.

Burrows-Wheeler Aligner (BWA 0.7.8)^7^ was used to map sequencing reads against a reference RNA database compiled from the gene sequence data downloaded from Microbial Genome Database for Comparative Analysis (mbgd_2013–02)^49^, Feb. 2009 assembly of the human genome (hg19, GRCh37 Genome Reference Consortium Human Reference 37 (patch 13)) and virus records from NCBI GenBank (30/06/2014 version). Mapping statistics was obtained by SAMtools (version 0.1.19)^3^. Trost *et al.* reported the similarity of different groups of bacteria to the human proteome^50^. Although the similarity may be lower at RNA level, uncharacterized microbiome can have similar RNA to human’s. To preclude this possibility, reads mapped onto either bacterial or viral genes were searched by Basic Local Alignment Search Tool (BLAST)^51^ against hg19 mRNA database again at an e-value of 1e-10. Non-specific mapping results were not considered in further analysis.

An expression unit modifying from fragments per kilobase of transcript per million reads mapped (FPKM)^52^ was calculated to quantify gene expression. The unit was obtained by dividing the number of mappable bases per gene divided by gene length and the total number of mappable bases per sample. Gene length information was obtained from the reference transcriptome. Human, bacterial, viral, and fungal RNA expression was derived by summing the total number of mapped bases in a particular domain (or human) per sample. The RNA expression ratios between human to bacteria (RNA human/bact), human to viruses (RNA human/virus) and human to fungi (RNA human/fungi) could then be calculated.

Hierarchical clustering with multiscale bootstrap resampling was done by pvclust 1.3–2^53^ for the bacterial genus RNA expression unit matrix by different samples. Correlation coefficient and average linkage were used and p-values were computed for each of the clusters. Since *Escherichia (E.) coli* gene expression was abundant, *E. coli* genes were selected as the background in the DAVID Gene Functional Classification Tool^8^ to investigate which gene functional categories were enriched. The gene mapping results from iPOP and LVAD studies were also searched against CAZy by dbCAN (15/03/2014 version)^54^. Keywords “glucose”, “lipopolysaccharide”, “O-antigen” and “lipid A” were used to search the number of glucose- and lipopolysaccharide-related genes expressed at a particular time point. Microbiome analysis was done by vegan 2.1–40^4^. Different models of Rank-Abundance Dominance at genus level were fit according to the Akaike Information Criterion to study the change in bacterial community richness and evenness.

**Figure 4:**
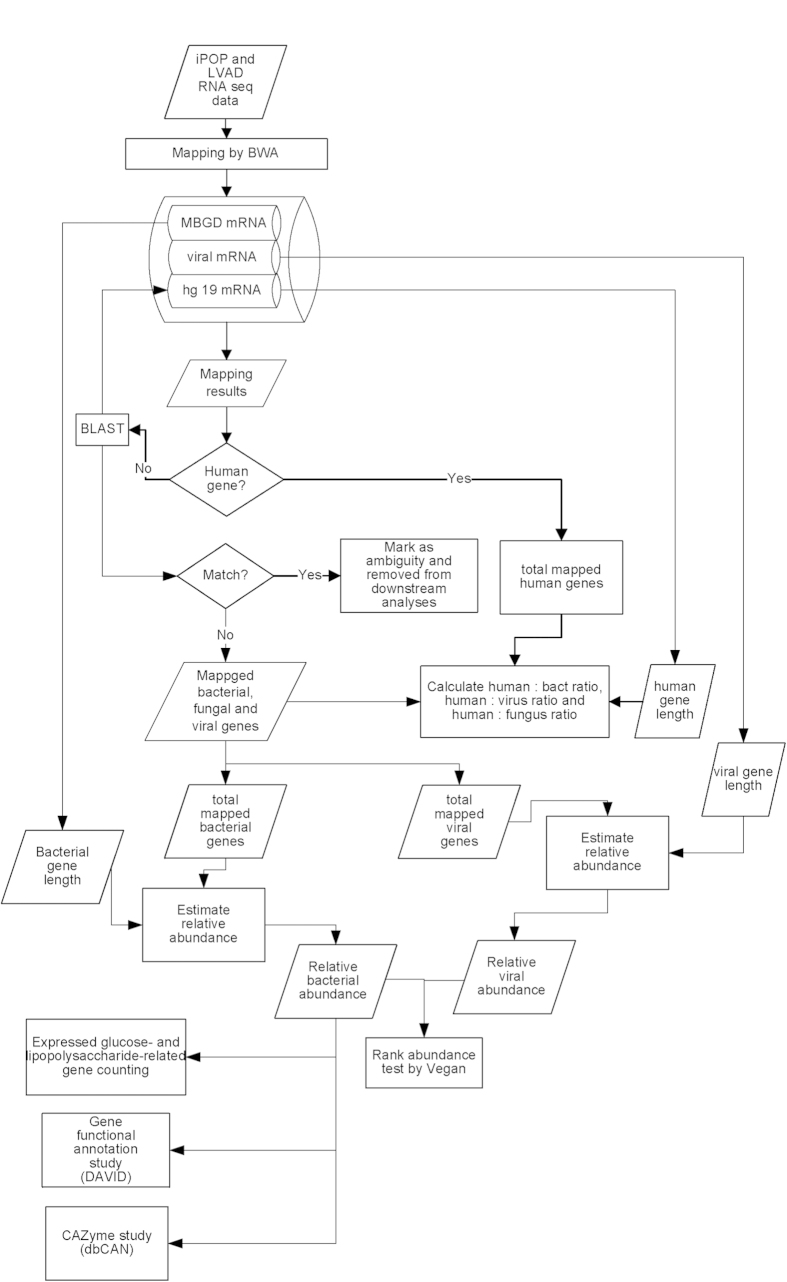
A schematic diagram summarizing the data analysis workflow in this study.

To ensure our pipeline can perform sensitive and specific mapping, we employed a shotgun sequence simulator, Grinder^55^ (http://sourceforge.net/projects/biogrinder/) to generate two sets of artificial human, bovis, mouse and yeast transcriptome data under 0 or 2 mismatch allowance. The human, bovis and mouse reference gene sequences were retrieved from ftp://ftp.ncbi.nih.gov/genomes and yeast’s from The Saccharomyces Genome Database^56^. These options were based on Wang *et al.*’s study, which evaluated the content of non-human RNA in blood components taking sequencing and mapping errors into consideration^24^. Since high RNA human/bact ratio was identified for five consecutive time points after RSV infection, we also conducted a permutation test of the 20 time points to count the occurrence of consecutive time points after random shuffling to evaluate whether the high RNA human/bact ratio observed was due to artifacts or contamination. The data workflow of this study is depicted in Fig. 4.

## Additional Information

**How to cite this article**: Leung, R. K.-K. and Wu, Y.-K. Circulating microbial RNA and health. *Sci. Rep.* **5**, 16814; doi: 10.1038/srep16814 (2015).

## Supplementary Material

Supplementary Information

Supplementary dataset 1

Supplementary dataset 2

Supplementary dataset 3

Supplementary dataset 4

Supplementary dataset 5

Supplementary dataset 6

Supplementary dataset 7

Supplementary dataset 8

Supplementary dataset 9

Supplementary dataset 10
