# Supplementary material for: Circulating microbial RNA and health
Source: Sci Rep. 2015 Nov 18;5:16814. doi: 10.1038/srep16814 (PMC4649493; doi:10.1038/srep16814)
Supplement: Supplementary Information [file srep16814-s1.pdf]

Circulating microbial RNA and health

Ross Ka-Kit Leung and Ying-Kit Wu

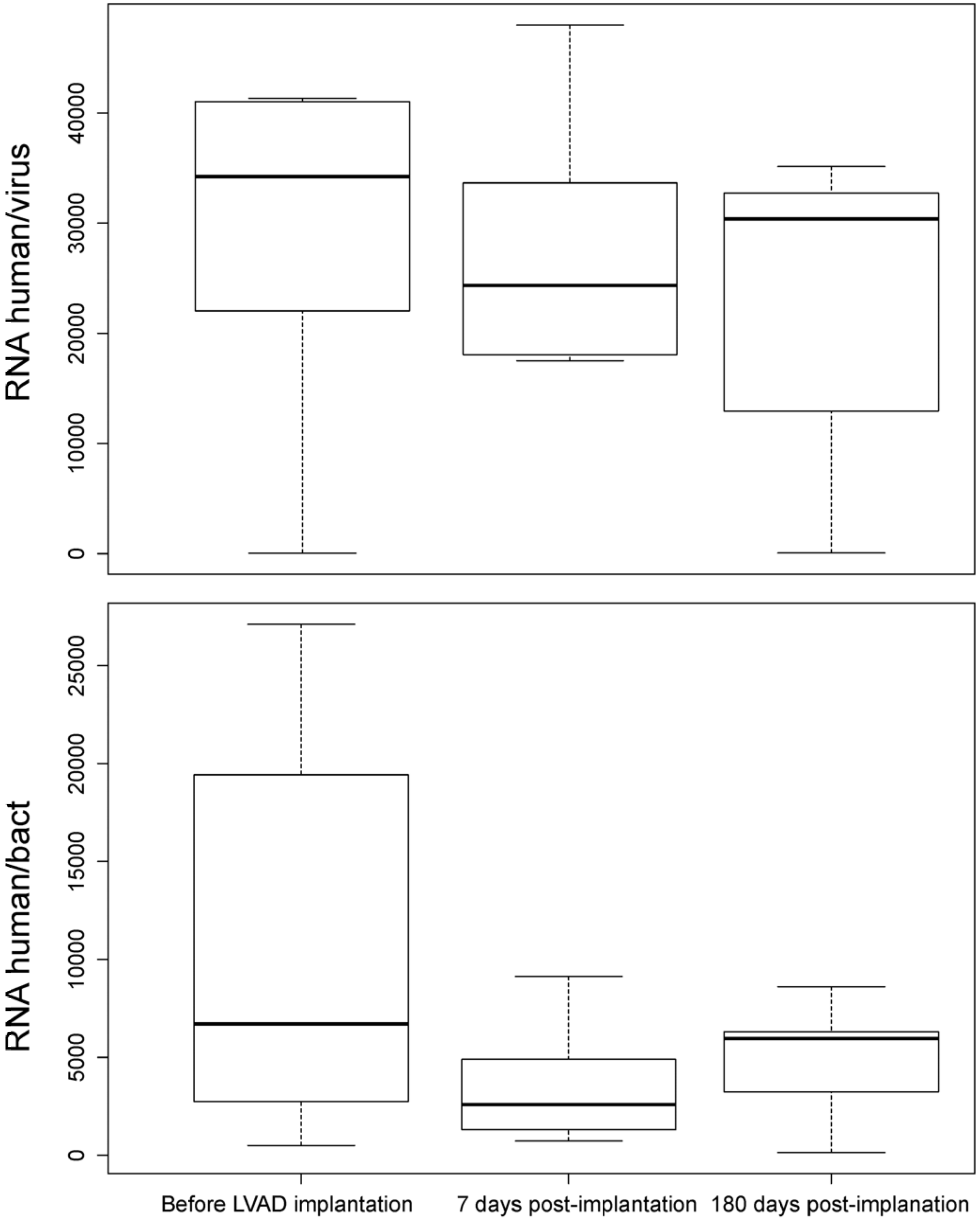

Supplemental Figure 1. Plots of RNA expression ratios of human to bacteria and human to virus at different time points for the nine end stage heart failure patients
